# Does seed size mediate sex-specific reproduction costs in the *Callosobruchus maculatus* bean beetle?

**DOI:** 10.1371/journal.pone.0225967

**Published:** 2019-12-12

**Authors:** Dariusz Krzysztof Małek, Maciej Jan Dańko, Marcin Czarnoleski

**Affiliations:** 1 Institute of Environmental Sciences, Jagiellonian University, Kraków, Poland; 2 Max Planck Institute for Demographic Research, Rostock, Germany; Universidade de São paulo, BRAZIL

## Abstract

There is a trade-off between reproductive effort and adult longevity, and when resource allocation is taken into account, it is especially pronounced in species that have aphagous adult forms. This trade-off may be further complicated by environmental factors such as nutrient availability during larval development and by the other sex, which influences the costs of reproduction due to the presentation of nuptial gifts. Here, we examined the influence of larval nutrient quantity on the sex-specific longevity costs of reproduction in the gift-giving seed beetle *Callosobruchus maculatus*. We found no indication that differences in the nutrient quality of larger and smaller host seeds influence survival in virgin and reproducing individuals or nuptial gift size in reproducing individuals. However, in the case of reproducing individuals, the effect of seed size on survival was statistically marginal. Therefore, we advise taking this into account when investigating reproductive efforts in this species. We have also observed interesting interactions between male and female reproductive costs. While females had generally higher mortality than males, nuptial gifts resulted in lowered female mortality and increased male mortality. Additionally, we found a possibly non-linear relationship between nuptial gift size and the offspring production rate of female recipients.

## 1. Introduction

The costs of parental investment to current offspring production negatively affect growth, future reproductive capacity, and lifespan [1,2,3]. The link between reproductive effort and survival is believed to be rooted in an allocation trade-off that arises under conditions of resource limitation in such a way that resources channelled to the production of offspring reduce the pool of resources available for the maintenance of parental tissues [4,5,6]. Males and females also incur different reproductive costs [7]. Although such differences originate from differences in the investment in individual gametes [8], the total costs of gamete production may be similar for both sexes [9], and a reliable assessment of the ultimate reproductive costs should consider a range of sex-specific behaviours and physiological activities [8].

The reproductive costs of mating partners can also influence one another, especially if mating involves the transfer of resources between partners. These so-called nuptial gifts can take very different forms and aim to improve donor fitness [10], and their production is thought to involve strong sexual selection [11]. At the same time, the recipient of nuptial gifts can either mutually benefit from a nuptial gift or be exploited by the effects of the gift [11]. According to Voigt et al.[12], katydids are an extreme example with females theoretically being able to survive exclusively on gifts provided by males. Some types of gifts are more useful for empirical investigation of life history trade-offs than others [10]. For example, an endogenous gift that is manufactured or sequestered by a donor is especially costly in aphagous species, which cease feeding after completing development and maturation. Reproductive costs are also sensitive to living conditions. This offers an opportunity to address the environmental dependence of energy budgets and life history trade-offs [3,13]. For example, virgin individuals were shown to live longer than mated individuals when the food supply was somehow restricted (e.g., [14]). Evidence for fruit flies and scorpionflies suggests that food supply also affects the size of nuptial gifts produced by males and the choosiness of gift-receiving females towards their mates. Under nutritional stress, males were observed to limit nuptial gifts offered to female partners, either by producing smaller or fewer gifts (endogenous gifts in fruit flies; [15]) or by partially consuming the gift before offering it to the female (exogenous gifts in scorpion flies; [16]). Under nutritional stress, females of *Drosophila subobscura* were more selective towards well-fed males that could produce larger endogenous gifts [15]. In contrast, protein-deprived females of the Mediterranean fruit fly *Ceratitis capitata*, which do not receive nutptial gifts from their mates, were relaxing their selectivity of partners, accepting matings with smaller males, which are otherwise typically rejected by well-fed females [17].

**Fig 1:**
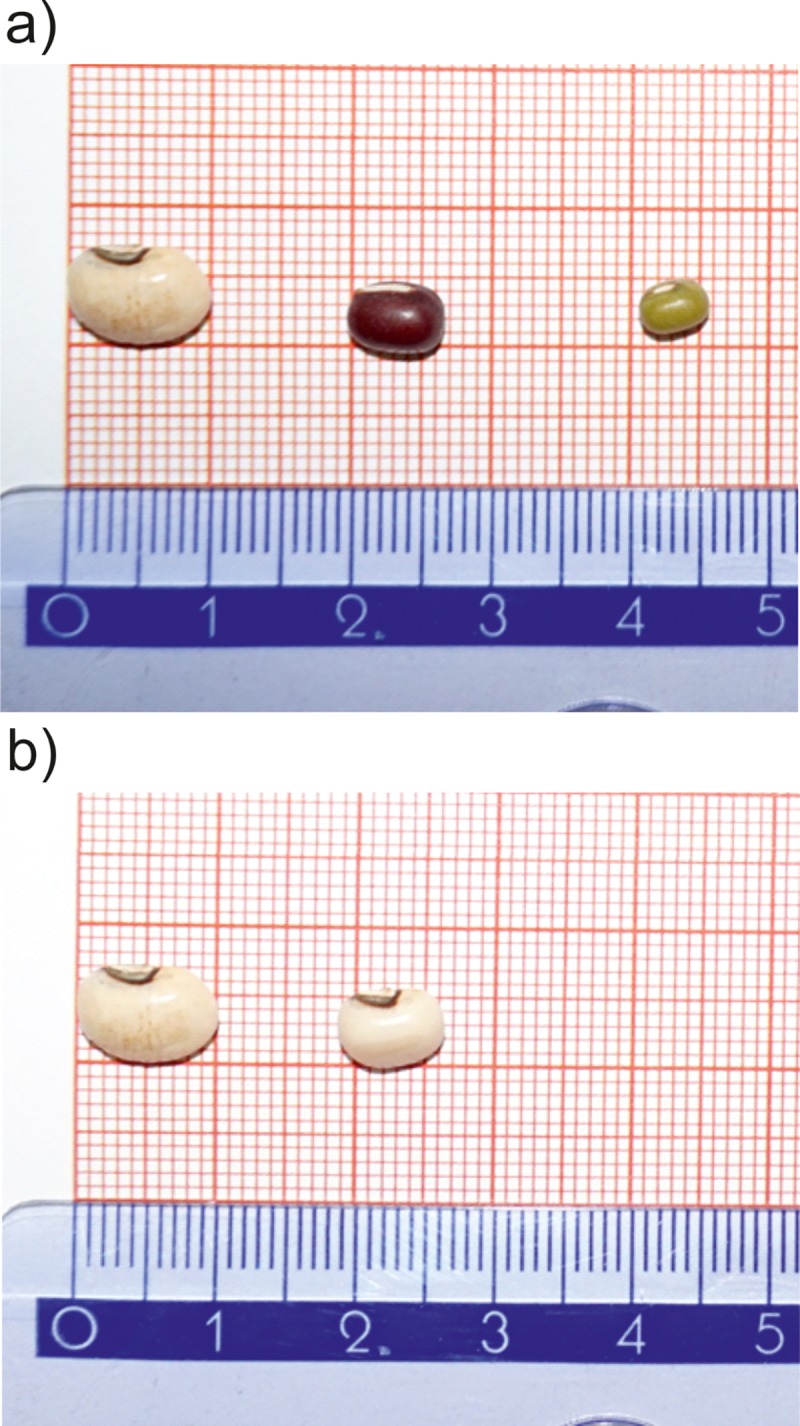
Bean size comparisons. a) Interspecies differences in Fabaceae seeds used to culture C. maculatus: (from left) 1 black-eyed pea (*Vigna unguiculata*, cowpea), 2 adzuki bean (Vigna angularis), 3 mung bean (Vigna radiata). b) Intraspecies differences in seed size in cowpeas.

Addressing effects of food supply on nutptial gifts and their fitness effects, we studied the *Callosobruchus maculatus* bean beetle, a cosmopolitan pest of stored *Fabaceae* seeds. We explored the life history effects of endogenous nuptial gifts (provided by males as an ejaculate during copulation) and their dependence on developmental conditions of the larval stage of *C*. *maculatus*. Adults of *C*. *maculatus* are regarded as faculatively aphagous, surviving and reproducing without access to food and water but being able to utilize resources if they are available. Seminatural environment of seed beetles created by facilities of bean seed storage as well as laboratory experiments on seed beetles typically do not provide food and water to adult *C*. *maculatus* [13]. Under these conditions, the physiological state of reproducing *C*. *maculatus* strongly depends on the amount of resources that were consumed by larvae during development, which, for these beetles, takes place inside a bean seed [18]. Developmental conditions are likely to have a stronger impact on the physiological performance of males than that of females because male *C*. *maculatus* produce large ejaculates that constitute nuptial gifts and sperm, which are transferred to females upon copulation [19]. Importantly, earlier studies of *C*. *maculatus* demonstrated conflicting evidence of the effect of nuptial gifts on female fecundity and survival [20,21]. As a result, it is difficult to conclude whether nuptial gifts benefit males by increasing their chances of paternity or their female partners by increasing temporal and/or lifetime offspring production. There is evidence suggesting that developmental conditions inside seeds can affect the reproductive performance of adult *C*. *maculatus*. Martinossi-Allibert et al. [22] found that stressful conditions (thermal stress and suboptimal host species) resulted in reduced fitness but this effect was stronger for males than females. Additionally, sexes reacted differently to particular host species, with lifetime reproductive success in females tending to be more sensitive to development on adzuki beans and males to development on mung beans. Fox et al. [21] also developed *C*. *maculatus* larvae in seeds originating from different *Fabaceae* species and demonstrated that the size of nuptial gifts from males depended on the plant species. However, the direction of this effect was dependent on the origin of the population used. *Fabaceae* species are known to deposit different types of secondary compounds in their seeds [23], which might account for the results from Fox et al. [21]. Different populations may be better adapted to a specific host species more common in their place of origin. However, an alternative and not mutually exclusive explanation is that seeds from different plant species differed in mass and thus in the amount of resources available to larvae during development. Indeed, seed sizes differ significantly between *Fabaceae* species (Fig 1A) and are also highly variable within species (Fig 1B). Certainly, it is widely known that multiple larvae of *C*. *maculatus* can complete development in a single bean seed, but such developmental conditions were shown to affect the life history traits of adults [24], indicating that the amount of resources in a seed can impose limitation for developing larvae. The results of such competition have also been shown to be dependent on the size of the bean seed (e.g. [25]). To address whether within-species differences in seed size correspond to measurable differences in adult size, the cost of reproduction and offspring production, we allowed *C*. *maculatus* to develop in seeds of variable mass obtained from the black-eyed pea *Vigna unguiculata*. If seed size is a limiting factor for *C*. *maculatus*, we expected that development in smaller seeds would result in reduced adult body mass and a shortened lifespan for both sexes. We also expected that development in smaller seeds would negatively affect the sizes of gifts produced by males and increase the longevity costs associated with gift production for males. For females, we predicted that development in smaller seeds would result in the production of fewer offspring. We also predicted that the size of the gifts received by females would positively affect their egg production and/or longevity, but we expected this effect to be more pronounced among females developed in smaller seeds, given that such females would be more restricted by resource availability.

## 2. Materials and methods

### 2.1 Experimental design

The laboratory culture of C. maculatus used in this study originates from beetles of the “Nigeria” strain, provided in the year 2014 through the courtesy of Professor Goran Arnqvist’s laboratory. To boost the number of individuals available for the experiment, animals in the culture were allowed to freely interbreed for over 20 generations under 27°C and 70% RH and a photoperiod 12 h:12 h (L:D). To complete the life cycle, the beetles were provided *V*. *unguiculata* seeds. Prior to the experiment, we randomly collected beans carrying one egg from the main culture, which prevented developing larvae to compete for resources within beans. This beans were kept separated until the eclosion of beetles. After this, fifteen freshly eclosed females were randomly selected from this pool. On the third day after eclosion, each female was paired for 24 h with one male. After removal of the males, females were provided unrestricted access to fresh *V*. *unguiculata* seeds for egg laying for 24 h. To examine the effects of seed mass on developing beetles, each female received seeds of varying sizes. After egg laying, each seed with a visible egg was weighed to the nearest 0.001 mg on a microbalance (Mettler-Toledo XP26, Mettler-Toledo GmbH, Laboratory & Weighing Technologies, CH-8606 Greifensee, Switzerland) and placed in a separate Eppendorf tube with a perforated lid. Please note that in this study we use seed mass as a proxy of seed size, this is dependent on an assumption that all seeds are of the same density. The tubes containing eggs were placed in standard conditions for the completion of beetle development. Note that our egg collection procedure ensured that all eggs used in the experiment were produced by females of equal age. Earlier studies of *C*. *maculatus* demonstrated the significant effect of female age at egg-laying on offspring longevity [26]. Upon eclosion, individuals in the new generation of beetles (males and females) were allocated to one of two reproductive treatments: reproducing or non-reproducing individuals. Note that the progeny of each female that laid eggs in our experiment were uniformly allocated to each treatment, and males and females from each family were evenly allocated to each treatment. Beetles allocated to the reproducing group were paired for a single copulation on the third day after eclosion. We avoided pairing individuals from the same mother. After copulation, each partner was placed in a separate container, following which all experimental animals were individually maintained until death. Females from the reproducing group were provided with bean seeds ad libitum for egg laying.

In total, our experiment involved 111 individuals (53 females and 58 males). Male *C*. *maculatus* beetles emerge from their host seeds with only partially filled seminal vesicles [27]. It is therefore possible that differences in their ability to produce complete spermatophores could obscure the relationship between gift size and the number of progeny produced by the female recipient [28]. To avoid this possibility, we ensured that all animals involved in the measurements (males and females from all treatments) were of the same age (3 days ±12h) at the beginning of the experiment. For each experimental beetle, we measured its adult body mass and longevity (days). Immediately after eclosion, each adult’s mass was determined to the nearest of 0.001 mg on a microbalance (Mettler-Toledo XP26, Mettler-Toledo GmbH, Laboratory & Weighing Technologies, CH-8606 Greifensee, Switzerland). In the reproducing group, we also assessed the mass of the nuptial gifts and the reproduction rate of the females. The nuptial gift mass was estimated by weighing males before and after copulation. The loss of body mass following copulation was our proxy of the absolute mass of the nuptial gift [29] but we stress here that it needs to be further investigated which portions of the ejaculate comprise sperm cells, seminal fluids and substances allocated as a nuptial gift (see Discussion). To control for the effects of body size, we also calculated the relative nuptial gift size by dividing the absolute value of the nuptial gift by the body mass of the male prior to copulation. For reproducing females, we counted the number of progeny produced during the lifespans of females that survived to the adult stage.

### 2.2 Statistical analysis

Statistical analyses were performed with the *R environment* software *[30]*. Because our experimental animals originated from fifteen different females, all models considered the effect of different female parents as a random factor.

#### 2.2.1 Adult size and gift size: The effects of seed size

To test our hypothesis that adult body mass depends on the seed size and the sex of the beetle, we applied a linear mixed (random intercept) model. The model included adult body mass as the dependent variable and sex and bean seed mass as fixed factors, and the mother’s identity was used to estimate random effects. We used the same method to test whether the size of nuptial gifts produced by reproducing males depends on body mass and the size of seeds from which the males originated. The model included nuptial gift size as a dependent variable and male body mass and seed size as fixed factors. This analysis was performed with both the absolute and relative nuptial gift measurements using the maximum likelihood method. All non-significant interactions were removed from the models. P-values were calculated using the Wald chi-square test. The model for adult body mass and absolute nuptial gift size assumed normally distributed errors, whereas the model for the relative gift size used a beta distribution [31]. The fit of each model was assessed by analysing the residuals.

#### 2.2.2 Longevity: The effects of seed size, reproduction and nuptial gifts

To test our hypotheses regarding the effects of seed mass on longevity, we performed two survival analyses that explored data on either all experimental animals or reproducing individuals only. Our experimental design included a mixture of fixed categorical factors (sex and reproductive treatment), fixed continuous factors (body mass, seed mass, gift size), and a random effect (identity of the mother). Therefore, for each type of analysis, we used the following model selection procedure. For survival analysis, random effects are typically modelled by frailty terms describing hidden differences in the survival of individuals in a population [32,33]. In our data, each group of individuals shared the same hidden frailty (progeny from the same mother); consequently, the parametric proportional hazard model with shared frailty was used [33,34,35]. In the proportional hazard model, setting a frailty term proportionally affects the hazard function. This assumption was visually tested by inspecting the log cumulative hazard (log(-log lx) plots, where lx indicates survivorship) for different categorical variables. For continuous variables, a semi-parametric approach based on the Cox proportional hazard frailty model was used, and Schoenfeld residuals were inspected for departures from linearity (survival R package, [36,37,38]). Both tests suggested that the proportional hazard assumption held. The parametric frailty model needed to include assumptions about baseline hazard and frailty distribution. A linear relationship between log cumulative hazard and log survival time in the early stages of the curves (where the differences in frailty have a negligible effect) suggested a Weibull distribution. This was further explored by fitting a series of basic models (*parfm R package*, [39]) that all included sex, treatment, seed mass, adult body mass and sex × treatment interaction terms but used different combinations of baseline hazard (Gompertz, Weibull and exponential) and frailty distribution (none or gamma). The model selection was based on the Akaike information criterion (AIC; [40]). The results supported the choice of a Weibull baseline hazard and the presence of differences in the frailty modelled by a gamma distribution.

The selection of the most parsimonious model was performed by a progressive hierarchical likelihood ratio test (hLRT). The method started from the basic model, which included all main effects (sex, treatment, seed mass, and adult body mass) and the sex × treatment interaction (already checked for significance). In the next iterations, we tested for the possibility of further extension by sequentially adding a single 2-way interaction and performing a likelihood ratio test. The algorithm was repeated until no new interactions could be added. We found that the basic model could not be improved. The same procedure was applied to the reproducing individuals to choose the most parsimonious model to describe the effect of nuptial gift size on survival. The initial model included all main terms: sex, bean size, adult body mass, and gift size. The model selection extended this by including the sex × gift size interaction.

The fits of the selected models were validated by checking trends in the martingale residuals [41] and by estimating variance inflation factors [42,43] (see S1 File). To visualize and interpret the model predictions, we used the ideas of marginal hazard and conditional marginal hazard. The marginal hazard is calculated from the estimated model. It is the mean hazard over all individuals in the considered group with respect to differences in frailty. The conditional marginal hazard is the marginal hazard assuming one of the continuous covariates is fixed for all individuals (see S1 File for details). For the sake of simplicity, the term “hazard” is used interchangeably with mortality throughout the paper.

#### 2.2.3 Female reproduction: The effects of seed size and nuptial gifts

The hypothesis that the number of offspring produced by mated females is affected by the weight of the nuptial gifts and the seed mass was tested by fitting a generalized additive mixed model [37] to the data on the number of progeny produced over a female’s lifetime. The model included seed mass, adult female mass, and the size of the received gift as fixed factors and the identity of the female’s mother as a random effect (random intercept model). The gift size was assumed to act non-linearly on progeny number and was modelled as a smooth term [44]. The model assumed a negative binomial distribution and log link function. We analysed two versions of the model, with and without an offset equal to the log of the female lifespan. Note that the model that did not consider female lifespan compared the total lifetime offspring production between females, whereas the model that accounted for females’ longevity is equivalent to comparing the mean offspring production rates between females.

## 3 Results

### 3.1 Adult size and gift size: The effects of seed size

**Fig 2:**
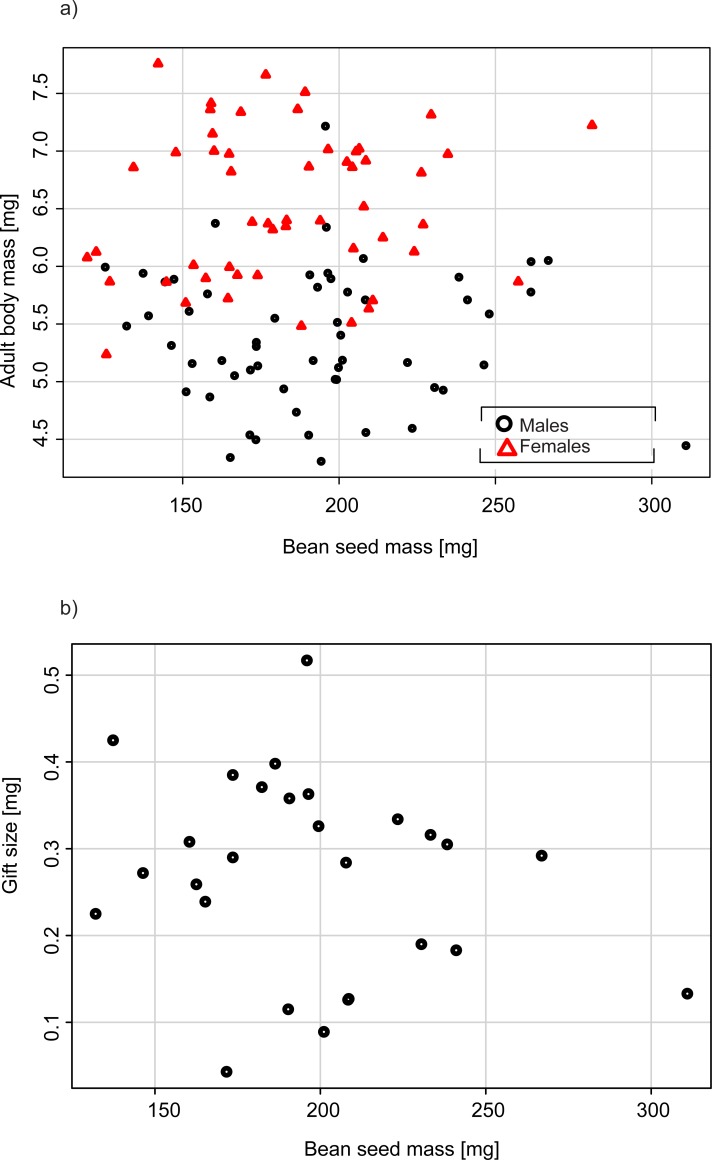
Effects of bean seed size on a) adult body size and b) nuptial gift size in *C*. *maculatus*.

**Fig 3:**
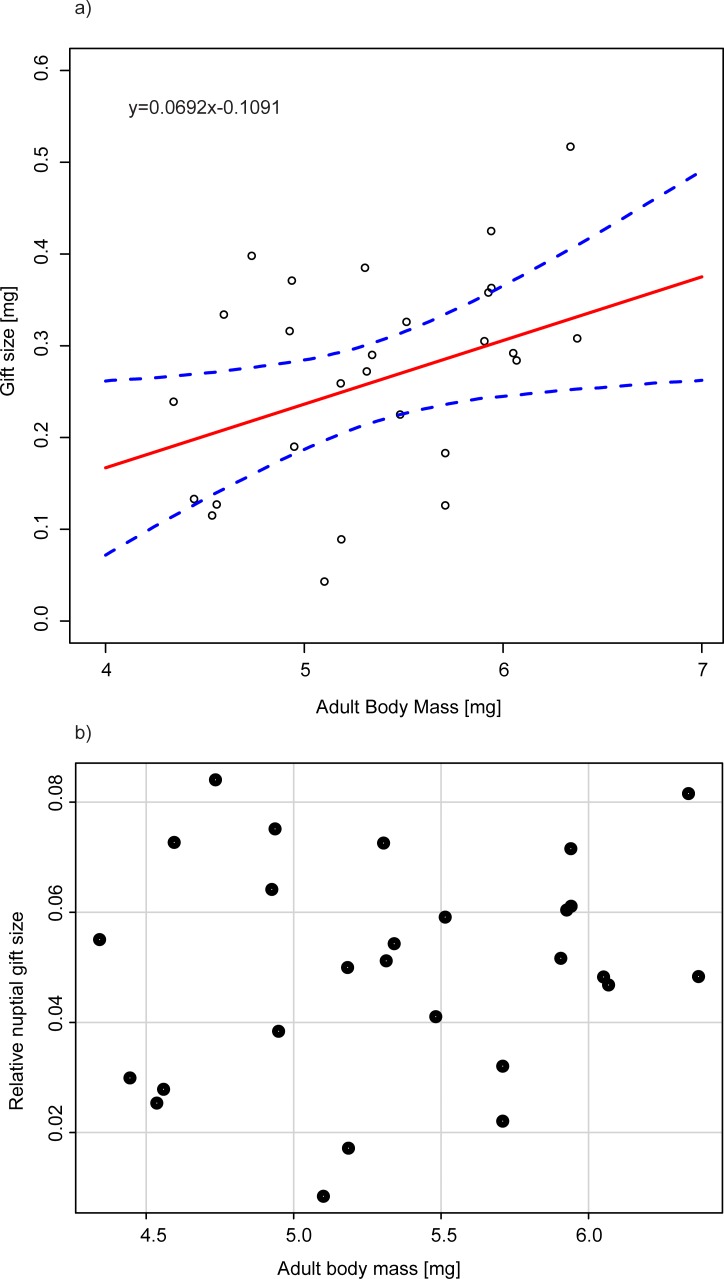
**Effect of adult body size of *C*. *maculatus* on a) absolute and b) relative nuptial gift size.** Dashed lines represent 95% bootstrapped confidence intervals for fixed effects with excluded residual variance calculated using MerTools [45]. See Table 1B and 1C.

Adult females were significantly larger than males (p<0.001), and seed mass did not affect adult body mass (Table 1A, Fig 2A). Seed mass also did not affect the size of the gifts produced by males (Table 1B, Fig 2B). Large males produced larger gifts (p = 0.029, Table 1B, Fig 3A), but the relative mass of the gifts did not change with the body mass of the male (Table 1C, Fig 3B), indicating that gifts constituted a constant proportion of the male body mass.

**Table 1:** Wald chi-square test results of mother-specific random intercept models for adult body mass (a), absolute nuptial gift size (b), and the proportion of the male body mass used for the nuptial gift (relative nuptial gift size) (c) in *C*. *maculatus*.

a) adult body mass				
	ChiSq	Df	P	
**Sex**	**98.936**	**1**	**<0.001**	
**Bean size**	**0.257**	**1**	**0.6124**	
b) absolute nuptial gift size				
	ChiSq	Df	P	
**Adult body mass**	**4,797**	**1**	**0.029**	
Bean size	0.810	1	0.368	
c) proportional nuptial gift size				
	Estimate	Std. Error	z value	p
(Intercept)	-3.184	0.916	-3.477	<0.001
Adult body mass	0.094	0.146	0.643	0.520
Bean size	-0.002	0.002	-0.808	0.419

### 3.2 Longevity: The effects of seed size, reproduction and nuptial gifts

**Fig 4:**
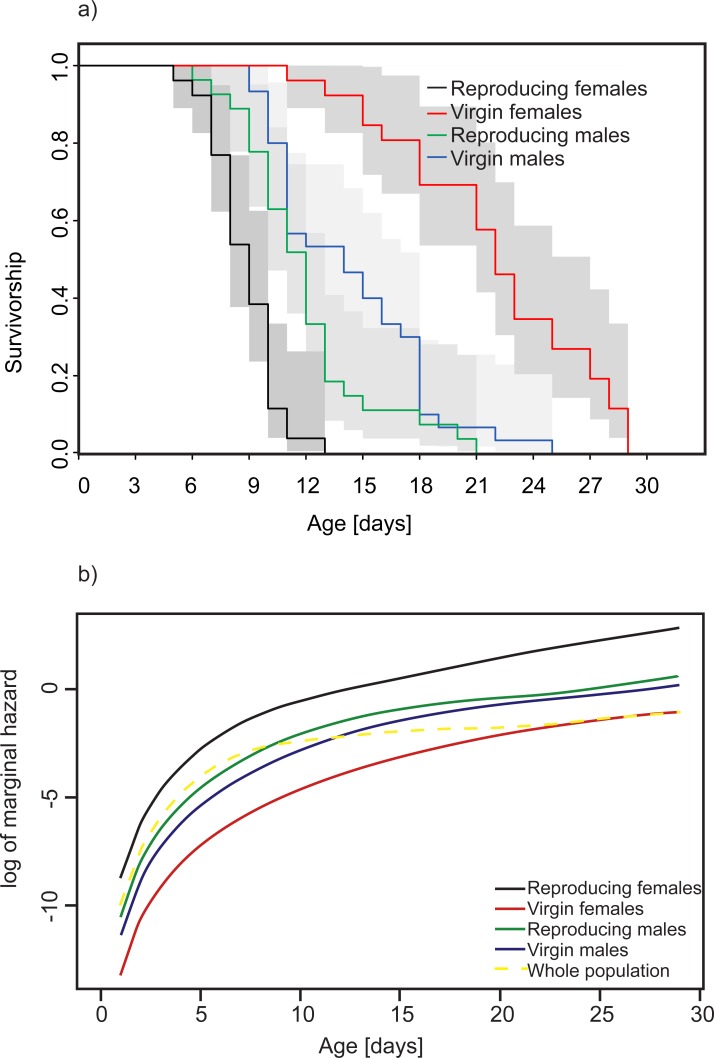
Effect of reproduction on survival in *C*. *maculatus*. **a) Observed survival of experimental animals.** b) Marginal hazard for all experimental treatments predicted by our model. The marginal hazard is the mean hazard over all individuals of the considered group with respect to differences in frailty (random factor) calculated from the estimated model (see Table 2).

**Fig 5:**
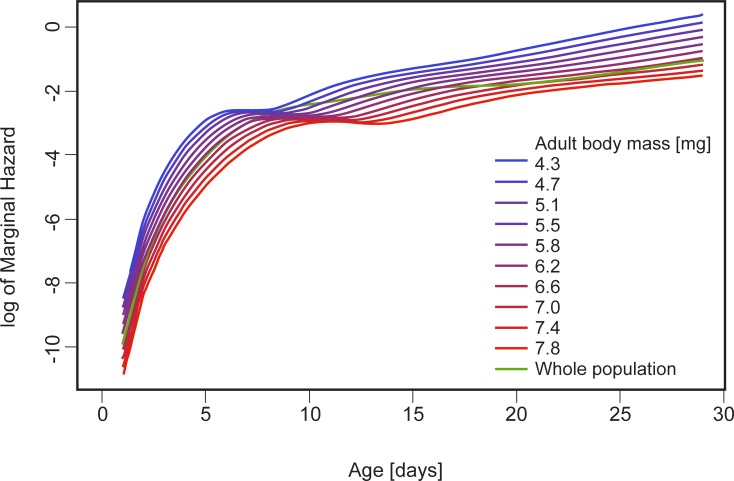
Predicted effect of adult body mass on marginal hazard in *C*. *maculatus* (conditional marginal hazard—marginal hazard assuming one of the continuous covariates to be fixed for all individuals). See Table 2.

We found a significant effect of sex, reproductive treatment, adult body mass, and the combination of sex and reproductive treatment on survival patterns (p<0.001 in all cases, Table 2); however, there was no significant effect of bean mass on survival (p = 0.73, Table 2). In general, the highest mortality was observed in reproducing females, and the lowest mortality was observed in virgin females, whereas reproducing and virgin males exhibited roughly similar mortalities to each other (slightly higher in reproducing males) in between those of the reproducing and virgin females (Fig 4). Additionally, large beetles survived longer than smaller beetles (Fig 5) regardless of sex and treatment (p<0.001, Table 2).

**Table 2:** The parametric frailty model assuming Weibull distribution and gamma frailty for all individuals of *C*. *maculatus*.

	Estimate	Std. Error	p
Theta (frailty par.)	0.1572	0.1290	
Rho (Weibull shape par.)	4.7296	0.3877	
Lambda (Weibull scale par.)	0.0026	0.0036	
**Sex (Male)**	**-2.8240**	**0.4835**	**<0.001**
**Treatment (Virgin)**	**-4.5469**	**0.4850**	**<0.001**
Bean size	0.0011	0.0031	0.7263
**Adult body mass**	**-0.6894**	**0.1998**	**<0.001**
**Interaction (sex × treatment)**	**3.8895**	**0.5384**	**<0.001**

**Fig 6:**
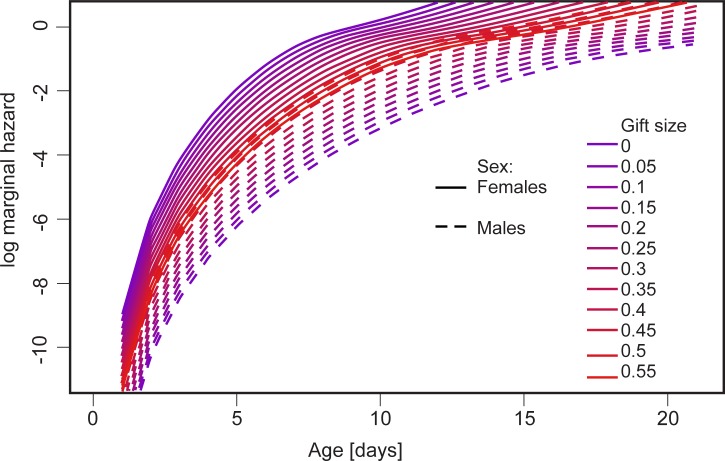
Predicted effect of nuptial gift size on marginal hazard in both sexes of *C*. *maculatus*. See Table 3.

The analysis of the survival of reproducing individuals showed significant effects of sex (p<0.001, Table 3) and the combination of sex and gift size (p = 0.006, Table 3). The effect of seed size was close to significance (p = 0.082, Table 3), with individuals developing in smaller seeds surviving for shorter lengths of time. The combination of sex and gift size is shown as a marginal mortality in Fig 6. Females had generally higher marginal mortality than males, but larger gifts resulted in lowered female mortality and increased male mortality up to the point at which male mortality became higher than female mortality.

**Table 3:** The parametric frailty model assuming Weibull distribution and gamma frailty for reproducing *C*. *maculatus*.

	Estimate	Std. Error	p
Theta (frailty par.)	0.2558	0.2314	
Rho (Weibull shape par.)	5.4213	0.7059	
Lambda (Weibull scale par.)	0.0009	0.0021	
**Sex (Male)**	**-4.8499**	**1.0594**	**<0.001**
Bean size	-4.2520	2.4488	0.0825
Adult body mass	-0.0045	0.0051	0.3764
Gift size	-0.4326	0.2837	0.1272
**Interaction (sex × gift size)**	**8.5111**	**3.1087**	**0.0062**

### 3.3 Female reproduction: The effects of seed size and nuptial gifts

**Fig 7:**
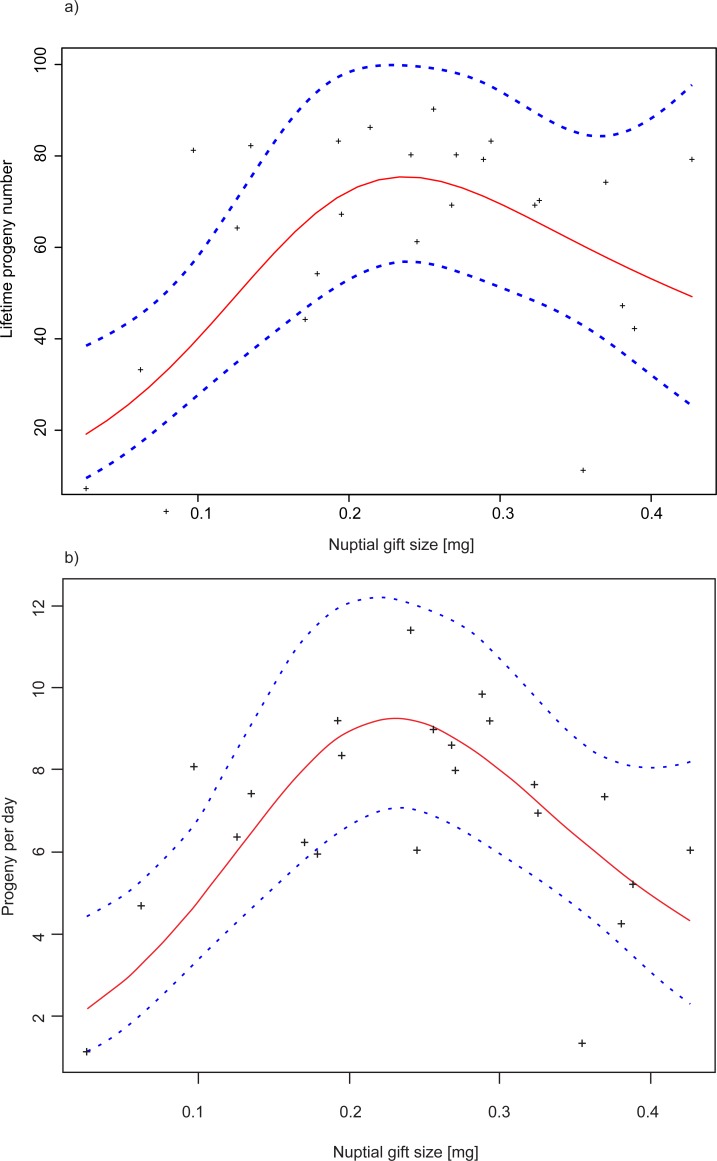
Effect of nuptial gift size on reproduction of female *C*. *maculatus*. a) Lifetime progeny production b) Mean daily reproductive rate. See Table 4A and 4B respectively for details.

The analysis of reproductive performance of females reached similar conclusions whether we analysed total lifetime reproductive success or mean reproductive rates. Our analysis suggests a non-linear relationship between reproduction performance of females and the size of the nuptial gift received (see Fig 7A and 7B), as indicated by the significant effect of the smooth term for gift size in the generalized additive mixed model (p<0.001, Table 4A and 4B). For smaller gifts, the female reproductive performance increased with gift size, with the highest reproductive performance observed among females that received a gift of approximately 0.25 mg. After this point, the reproductive performance decreased with further increases in gift size. The bean mass (p = 0.173, Table 4A; p = 0.296, Table 4B) and female adult body mass (p = 0.120, Table 4A; p = 0.192, Table 4B) did not affect reproductive success.

**Table 4:** Generalized additive mixed models for a) the lifetime number of progeny b) the mean daily reproductive rate of reproducing females in *C*. *maculatus*. Both models assumed a negative binomial distribution and log link function; the model for b) also considered an offset equal to the log of the female lifespan.

a) Model for the lifetime number of progeny Parametric coefficients:				
	Estimate	Std. Error	z value	p
(Intercept)	1.6312	1.2330	1.323	0.186
Bean size	3.9453	2.8982	1.361	0.173
Adult body mass	0.2507	0.1614	1.553	0.120
Approximate significance of smooth terms:				
	edf	Ref. df	ChiSq	p
**Nuptial gift size**	**2.304**	**2.669**	**10.12**	**0.00869**
b) Model for the mean daily reproductive rate Parametric coefficients:				
	Estimate	Std. Error	z value	p
(Intercept)	0.177664	1.171951	0.152	0.880
Bean size	0.002596	0.002759	0.941	0.347
Adult body mass	0.181916	0.153478	1.185	0.236
Approximate significance of smooth terms:				
	edf	Ref. df	ChiSq	p
**Nuptial gift size**	**2.42**	**2.762**	**13.17**	**0.0025**

## 4. Discussion

In our experiment, *C*. *maculatus* beetles developed in bean seeds that varied in mass from 119 to 310 mg, so some larvae had an almost three times larger supply of resources for development (Fig 2A). Nevertheless, we did not find evidence that this variance in seed size was related to detectable differences in adult size (Fig 2A, Table 1A), longevity (Table 2), the size of the gifts produced by males (Fig 2B, Table 1B and 1C) or the reproductive performance of females (Table 4). These findings do not support the idea that bean size affects resource availability at the larval stage, ultimately shaping the life histories of adult *C*. *maculatus*. In light of these results, the significant effects of seeds from different plant species on *C*. *maculatus* reported by Fox et al. [21] were likely related to species-specific differences in resource quality and the beetles’ abilities to assimilate them rather than differences in the amount of resources in seeds from different host species. Nevertheless, it would be premature to completely abandon the idea that seed size has fitness consequences in bean beetles. According to Cope and Fox [46], female *C*. *maculatus* beetles deposit eggs in seeds in a manner that maximizes the amount of resources available for each larva. In our experiment, larvae were forced to develop singly, but under natural conditions larvae are likely to face competition with conspecifics [47], and such competition would be reduced in larger seeds. The strength of the effects of seed size could be also context-dependent, which likely explains why we observed nearly significant positive effects of seed size on longevity in reproducing beetles (Table 3), which must become depleted of resources by the investment in nuptial gifts. Such an effect must be even more pronounced in natural conditions, where beetles are free to engage in multiple copulations, whereas in our experiment, we allowed only one copulation. In *C*. *maculatus*, the size of ejaculates decreases over consecutive matings [48] and this decrease becomes steeper as males invest more to the production of the first ejaculate [49], which indicates physiological burden imposed by multiple reproductive events. We conclude here that resolving to what extend seed size affects the life histories of wild bean beetles would require designing experiments that challenge the study animals with differently balanced demands and supplies of resources.

The potential effects of developmental conditions in seeds on future reproductive performance would certainly fit into the more general pattern of parental investments and their costs revealed by our study. We observed that among virgin individuals, females lived longer than males (Fig 4, Table 2), and in both sexes, larger individuals lived longer (Fig 5, Table 2). Also among reproducing individuals, either larger males or females survived longer than their smaller conspecifics, in agreement with earlier reports of Paukku and Kotiaho [50] for reproducing male *C*. *maculatus*. Although reproduction imposed significant costs for both sexes (Table 3), our results suggest that it might be generally costlier for females. In fact, reproducing females appeared to be the shortest living group, while virgin females were the longest living group (Fig 4). Overall, our findings agree with the common view that females suffer higher reproductive costs than males [8], also supporting evidence of some earlier studies on *C*. *maculatus*. For example, similar sex-related differences in *C*. *maculatus* survival were reported by Fox, Dublin and Pollitt [51], who found a higher initial mortality rate in females, but noted that male mortality was much rapidly increasing with age. Interestingly, results of selection experiment on *C*. *maculatus* suggest a sexual conflict over a fitness-maximising life strategy, resulting in the evolution of sub-optimally short lifespan in females and sub-optimally long lifespan in males [52]. The longevity costs of reproduction in female *C*. *maculatus* can take different forms. For example, after a single copulation, females with access to seeds for oviposition lived significantly shorter that virgin individuals, indicating physiological costs of reproduction [53]. Copulations in *C*. *maculatus* were shown to directly harm the body of females [54, 55]. Certainly, evaluations of the costs of reproduction can be imprecise if they are derived from the effects of a single copulation. Also, any comparison between the effects of a single copulation in males vs females would have to take into account the extent to which the lifetime reproductive success of each sex is determined by a single act of copulation. Although in *C*. *maculatus*, both females and males can have multiple partners [56,57], a single mating enables a female to lay eggs until death [20], while for a male each additional mating directly increases the chance to sire more eggs. Data for different species of seed beetles consistently demonstrated that reproductive success was more tightly linked to mating rate in males than in females [58].

If reproductive costs of one partner are modified by the reproductive involvement of the other partner, this relationship should become especially strong if partners exchange nuptial gifts. In our study reproducing males provided substantial ejaculates to their female partners. Following our estimations, ejaculates ranged from 0.06 to 0.31 mg, which amounted to up to 10% of the male’s body mass, suggesting significant investments of males to the production of ejaculates (our proxy of nuptial gifts). Competition between larvae that develop in seeds was shown to affect the size of ejaculates in *C*. *maculatus* [59], but this variance in ejaculate size was eliminated by our experimental design, though it must be considered under more natural conditions. According to our results, the production of nuptial gifts was apparently costly to male *C*. *maculatus*. When we compared males at a given body mass, we found that males with larger ejaculates lived shorter than males with smaller ejaculates (Fig 6, Table 3). Noteworthy, although larger males produced larger ejaculates, small and large males appeared to invest similar proportions of their body mass to ejaculates, which also agrees with some earlier results for *C*. *maculatus* [29]. Likely, such proportionality indicates that disproportionally large or small ejaculates would shift the balance between mortality costs and fertility benefits associated with different ejaculate sizes, but this hypothesis needs testing. It is interesting that Savalli et al. [60] showed that the pattern in the body size-dependence of ejaculate investments can vary between populations, though drivers of this variance await exploration. In three populations of *C*. *maculatus* studied by Savalli and colleagues, the mass of ejaculates consistently increased with male body mass, but in one population, ejaculates of large males formed a relatively smaller part of body mass compared to the ejaculates of small males. In the remaining two populations, similarly to our findings, ejaculates were scaling isometrically with male body mass. The evidence on the mortality costs of ejaculate production agrees with the idea that the resource budgets of male *C*. *maculatus* are limited by the facultative aphagy of this species [13]. Similarly, in Australian ground crickets from the genus *Pteronemobius*, an increased demand for nuptial feeding significantly lowered the male lifespan [61]. From the female perspective, a nuptial gift is of an either mutually beneficial or manipulative nature (using nomenclature from [11]), and it is not clear which of these two types of gifts evolved in *C*. *maculatus*. Here, we investigated both the longevity and the reproduction of females in relation to the size of the received nuptial gifts, predicting that a gift beneficial to a female would either increase its survival or reproductive success, or both traits. In accordance with this prediction, we found that larger nuptial gifts increased the survival and reproductive performance of their female recipients (Table 4). Interestingly, if we used our models to estimate values for even larger gifts, we found a theoretical gift size above which the mortality of males would be higher than that of females (Fig 6). Similarly to our findings, some earlier studies reported that ejaculate size was positively affecting the reproduction of females in *C*. *maculatus* and other seed beetle species (for example: [20,62]), but, there are also counter examples to this effect of nuptial gifts on females [21]. Ejaculates of virgin males of *C*. *maculatus* contain more sperm than a female can store in spermathecae [19], suggesting that a large female would be able to store more sperm and thus lay more eggs than a small female. Against this expectation, we found no relationship between reproductive success of females and their body mass (Table 4). A positive link between female body size and fertility is commonly observed in insects (e.g., [63]) and it was also demonstrated in an earlier work on *C*. *maculatus* [64]. Noteworthy, earlier studies typically did not differentiate between direct effects of nuptial gifts on the physiological capacity of females to lay eggs and indirect effects via the impact of nuptial gifts on females’ longevity and thus the duration of their reproductive life. In our study, conclusions about the effects of gift size on female reproduction were similar, whether we focused on the lifetime number of offspring or on the mean rate of offspring production. Nevertheless, future studies would benefit from measuring daily rates of egg-laying directly because the rate of offspring production was shown to decrease through time in female *C*. *maculatus* inseminated with single ejaculates [19].

Although we generally observed that nuptial gift size had a positive effect on female offspring production, this effect was diminishing with an increase in the size of ejaculates, and females that were receiving exceptionally large ejaculates (above ca. 0.25 mg) were characterised by a lowered reproductive performance. Therefore, if our results are representative, then the nuptial gift should be considered beneficial to the female rather than manipulative, but there seems to be a limit to how much the gift can increase the reproductive performance of the female recipient. In our experiment with single matings, a male donating an exceptionally large gift would equally suffer from poor reproduction of its female partner, but in more natural settings that promote remating, the large-gift males would likely outperform their small-gift rivals in sperm competition. Phylogenetic data for different species of seed beetles suggest that the intensity of female copulations with different partners imposed a selection pressure for the ejaculate investments of males, and these investments should be regarded as the means of combating sperm competition [49]. Indeed, there is growing evidence on the importance of postcopulatory sexual selection in *C*. *maculatus* driven by sperm competition [55,65] and perhaps also by cryptic selection of sperm by females [66,67]. This selective mechanism helps to understand coevolutionary trends among different seed beetle species in the size of ejaculates, characteristics of reproductive organs in males and females, and female fitness [48,65,68]. From the male perspective, larger ejaculates were suggested to delay the time of female remating and/or to help in replacing the sperm of rivals stored in spermathecae by own sperm [19]. Indeed, larger ejaculates decreased remating propensity of *C*. *maculatus* females [69] and manipulations of mating times in *C*. *maculatus* induced changes in the frequency of female’s remating [70]. From the female perspective, we hypothesise that by mating with large-gift males, a female of *C*. *maculatus* would not necessary aim at increasing her fertility, but would get a chance of acquiring “competitive genes” for her male offspring. This scenario has important implications for the evolution of mate choice among female *C*. *maculatus*–because on average larger males are donors of larger ejaculates, females would benefit from the preference for larger males. In support, Savalli and Fox [71] showed that larger *C*. *maculatus* males were more likely to win male-male competition for mates. Although this research did not find direct evidence that females simultaneously preferred larger mates, Fox and Moya-Laraño [72] observed that female *C*. *maculatus* preferred to mate with larger males, though this effect depended on a studied population.

The crucial insight from this work is that understanding of the evolution of nuptial gifts must incorporate context-dependence of the effects of gifts on longevity and reproduction of males and females. We envision that future studies should also consider that females might utilize different components of the received gifts for supporting survival and reproductive activity. For example, the aqueous component of the nuptial gift could be used by a female to increase its longevity, whereas nutrients could be spent for egg production. From the male perspective, this phenomenon would result in the allocation trade-off between these two pools of ejaculate resources. From the female perspective, a current state of female body would dictate the way in which a given ejaculate would be utilised to maximise female fitness. According to Fox and Moya-Laraño [72], female *C*. *maculatus* supplemented with food were less likely to remate, but the results of Ursprung et al. [73] and Edvardsson [74] suggest that remating depended more on water than on nutritional supply. If our hypothesis holds, using the total ejaculate mass as a proxy of the nuptial gift size would appear to be an oversimplifying approach to study the evolution of investments to nuptial gifts. A growing interest in the composition and nutritional value of ejaculates in seed beetles (e.g., [75,76]) promises that we will soon understand how, and whether at all, ejaculate components impact different components of female and male fitness.

## 5. Conclusions

• We did not find a significant effect of bean seed size on survival, but for reproducing individuals, the relationship was close to significance. We advise caution in future experiments and the use of bean seeds of a similar size. Especially, if there are multiple larvae per one bean as our experiment did not account for competition inside a bean seed.

• The absolute gift size increased with male body size, but the gift size was proportional to male body size.

• An increase in gift size resulted in the lowered mortality of females and the increased mortality of males up to a point at which male mortality was higher than female mortality. This suggests that the gifts are beneficial rather than manipulative for females.

• There is a possibly non-linear relationship between nuptial gift size and the rate of progeny production by females.

## Supporting information

S1 File Appendix. Definitions of the marginal and the conditional marginal hazard in the context of proportional hazard shared frailty models.
(PDF)

S2 File Original dataset. (CSV)
