# Supplementary material for: Does seed size mediate sex-specific reproduction costs in the Callosobruchus maculatus bean beetle?
Source: PLoS One. 2019 Dec 12;14(12):e0225967. doi: 10.1371/journal.pone.0225967 (PMC6907851; doi:10.1371/journal.pone.0225967)
Supplement: S1 File — Definitions of the marginal and the conditional marginal hazard in the context of proportional hazard shared frailty models. (PDF) [file pone.0225967.s001.pdf]

Appendix

Definitions of the marginal and the conditional marginal hazard in the context of proportional hazard shared frailty models

In the parametric proportional hazard shared frailty models the conditional hazard at age x of a subject i that belongs to the group g is defined as:

$$h_{g,i}(x) = u_g h_0(x|\rho) exp(X_{g,i}^T \beta),$$

where u_g is a frailty term in group g, h_0 is a parametric baseline hazard (e.g., the hazard of the Weibull distribution) at age x characterized by the set of parameters ρ (e.g. the shape parameter for the Weibull distribution), $X_{g,i}$ is a vector of observed covariates for subject i in group g, and β is a vector of corresponding regression coefficients. The frailty term u is realization of a random variable distributed according to a parametric probability density function (e.g., the gamma distribution characterized by variance θ and fixed mean of 1). In the paper to visualize the fitted models and make the model predictions we used the idea of marginal hazard [1]. The marginal hazard takes into account the composition of the considered cohort that changes with age. Particularly, it takes into account individual specific covariates X, estimated frailty u, and the fitted model depended on estimated parameters ($\hat{\beta}$, $\hat{\rho}$, and $\hat{\theta}$):

$$\bar{h}(x) = \frac{\sum_g \sum_{i \in g} h_{g,i}(x|u_g, X_{g,i}, \hat{\beta}, \hat{\rho}, \hat{\theta}) exp(-H_{g,i}(x|u_g, X_{g,i}, \hat{\beta}, \hat{\rho}, \hat{\theta}))}{\sum_g \sum_{i \in g} exp(-H_{g,i}(x|u_g, X_{g,i}, \hat{\beta}, \hat{\rho}, \hat{\theta}))},$$

where H is a cumulative hazard function:

$$H_{g,i}(x) = \int_0^x h_{g,i}(y) dy,$$

and $exp(-H_{g,i}(x))$ yields survivorship function. The marginal hazard can be also calculated for a subset (z) of individuals representing a combination of covariates (for example a combination of particular sex and treatment). Knowing that a given group g is unequivocally prescribed to a single individual i only, the marginal hazard for a subset of individuals z can be formulated as:

$$\bar{h}(x, z) = \frac{\sum_{i \in z} h_{g(i)}(x|u_{g(i)}, X_i, \hat{\beta}, \hat{\rho}, \hat{\theta}) exp(-H_{g(i)}(x|u_{g(i)}, X_i, \hat{\beta}, \hat{\rho}, \hat{\theta}))}{\sum_{i \in z} exp(-H_{g(i)}(x|u_{g(i)}, X_i, \hat{\beta}, \hat{\rho}, \hat{\theta}))}$$

A slightly different approach is applied to make predictions on continuous covariates (e.g. adult body mass, gift size). In such a case, one or more covariates in vector X are fixed and predictions on the marginal hazard are calculated conditionally to this fixed value(s). For example, if one want to make predictions for a particular value of gift size, then corresponding element of vector X should be substituted with the new value for each i.

The R [2] code to calculate the marginal and the conditional-marginal hazard is given below. The `marhaz.parfm` function returns a marginal hazard or a conditional marginal hazard for ages `x = 0, 1, 2, ..., max.x`. The argument `fit` is a object obtained by fitting a model using the `parfm` function of the `parfm` package [3], `data` is a data used to fit the model, `variable.name` and `variable.value` are the name and the value of the variable used to calculate the conditional marginal hazard, and `subset` is used for subsetting the `data`. The `marhaz.parfm` function calls the `predict.parfm` function of the `parfm` package to perform model predictions.

```r
marhaz.parfm <-function(fit, data, subset, max.x,
                        variable.name, variable.value = NULL){

  if (!inherits(fit, "parfm"))
    stop('The model must be fitted with the parfm function.')
  if (attributes(fit)$dist != 'weibull')
    stop('The function has been implemented only for the Weibull baseline so far.')
  if (missing(data)) data <- environment()
  if (!missing(subset)) data <- subset(data, subset)

  if(!grepl('Surv', attr(fit, 'formula'))) {
    warning('The model formula should be specified explicitly by using "Surv()."')
    f1 <- eval(parse(text=attr(fit, 'formula')))
    formula <- paste(f1[2L], f1[3L], sep='~')
  } else formula <- attr(fit, 'formula')

  # The step in x is set to one by default, if a change is needed than the
  # code for hazard calculation must be adjusted!
  xstep <- 1L
  if (missing(max.x)) {
    ZZ <- substr(formula, 1L, regexpr('~', formula, fixed = TRUE) - 1L)
    S <- eval(parse(text = paste('with(data = data,', ZZ, ')', sep = '')))
    x <- sort(unique(as.numeric(gsub(' ', '', gsub('+', '', S, fixed = TRUE)))))
    x <- seq(0L, max(x), xstep)
  } else
    x <- seq(0L, max.x, xstep)

  # Calculating Weibull baseline hazard
  rho <- fit["rho", 1L]
  lambda <- fit["lambda", 1L]
  baseline.haz <- lambda * rho * x ^ (rho - 1L)

  # Extract the model matrix. The intercept is stored in lambda,
  # it can be removed using [,-1L]
  mm <- model.matrix.default(as.formula(attributes(fit)$formula),
                             data = data)[, -1L]
  Terms <- colnames(mm)
  Terms2 <- attr(fit, 'terms')
  TermsX <- Terms2[which(!grepl(':', Terms2, fixed = TRUE))]
  if (length(Terms) != length(Terms2)) stop('Error in terms.')
  T2 <- splitstr(':',Terms2); T1 <- splitstr(':',Terms)
  if (!all(sapply(seq_along(unlist(T1)),
                  function(k) grepl(unlist(T2)[k],
                                    unlist(T1)[k],
                                    fixed = TRUE))))
    stop('Error in terms.')

  if (!missing(variable.name)) {

    if (!length(variable.value))
      stop(paste('Please give "variable.value" for', variable.name))

    if (length(variable.value) > 1L || length(variable.name) > 1L) {
```

```
      warning('Currently only one covariate can be substituted.')
      variable.name <- variable.name[1L]
      variable.value <- variable.value[1L]
    }

    ind.nam <- Terms2 == variable.name
    if (!sum(ind.nam))
      stop(paste('Unknown variable.name, use one of:  ',
      paste(TermsX, collapse = ', ')))
    mm[,ind.nam] <- variable.value
  }

  # Predicting X*Beta vectors for each individual
  coefi <- fit[, 1L]
  ind <- names(coefi) %in% colnames(mm)
  coefi <- coefi [which(ind)]
  M <- mm * matrix(coefi, dim(mm)[1L], length(coefi), byrow = TRUE)

  # Calculation of marginal hazard
  Pr <- parfm::predict.parfm(fit)
  Fr <- eval(parse(text = paste('data', '$',
                                attributes(Pr)$clustname, sep = '')))
  ui <- sapply(Fr, function(k) Pr[which(names(Pr) == k)])
  hazMat <- (ui * exp(rowSums(M))) %*% t(baseline.haz)
  cumhazMat <- t(apply(hazMat, 1L, cumsum))
  survMat <- exp(-cumhazMat)
  y <- colSums(hazMat * survMat) / colSums(survMat)
  y
}
```

Martingale residuals in the context of proportional hazard shared frailty models

The Martingale residuals are used to assess the goodness of the model fit. They are defined for each individual i as:

$$\hat{M}_i = \delta_i - u_{g(i)}\hat{H}_0(t_i)exp(X_i^T\beta)$$

where δ_i is the event indicator (0=right censoring, 1=death), and H_0 is the estimated cumulative baseline hazard. The calculation of Martingale residuals is performed by the `martingales.parfm` function (see below). It has only one argument, `fit`, which is a object returned by the `parfm` function of the `parfm` package [3].

```
martingales.parfm <- function(fit){

  if (!inherits(fit, "parfm"))
    stop('The model must be fitted with the parfm function.')
  if (attributes(fit)$dist != 'weibull')
    stop('The function has been implemented only for the Weibull baseline so far.')

  if(!grepl('Surv', attr(fit,'formula'))) {
    warning('The model formula should be specified explicitly by using "Surv()."')
```

```
    f1 <- eval(parse(text = attr(fit, 'formula')))
    formula <- paste(f1[2L], f1[3L], sep = '~')
  } else formula <- attr(fit, 'formula')

  Z <- substr(formula, 1L, regexpr('~', formula, fixed = TRUE) - 1L)

  # Exract a name of the data variable
  datan <- attributes(fit)$call$data
  if (length(datan) && nchar(datan)) {
    S <- eval(parse(text = paste('with(data = ', datan, ',', Z, ')', sep = '')))
  } else{
    stop('Name of the data variable must be given in the parfm function call!')
  }

  # Extract the survival data
  Time <- as.numeric(gsub(' ', '', gsub('+', '', S, fixed = TRUE)))
  Status <- as.numeric(!grepl('+', S, fixed = TRUE))

  # Check if the frailty is present
  include.frailty <- attributes(fit)$frailty != 'none'
  if (include.frailty){
    Pr <- predict.parfm(fit)
    Fr <- eval(parse(text = paste(datan, '$',
                                  attributes(Pr)$clustname, sep='')))
    FrailtyVec <- sapply(Fr, function(k) Pr[names(Pr)==k])
  } else FrailtyVec <- 1L

  #Calculate the Weibull baseline
  rho <- fit["rho", 1L]
  lambda <- fit["lambda", 1L]
  cum.baseline.haz <- lambda * Time ^ rho

  data <- eval(datan)
  mm <- model.matrix(as.formula(formula), data = data)[, -1L] # remove intercept
  coefi <- fit[, 1L]
  ind <- which(names(coefi) %in% colnames(mm))
  coefi <- coefi[ind]
  M <- exp(rowSums(mm * matrix(coefi, dim(mm)[1L], length(coefi),
                               byrow = TRUE)))
  resid <- Status - cum.baseline.haz * M * FrailtyVec
  resid
}
```

Variance Inflation Factors (VIFs) in the context of proportional hazard shared frailty models

The VIFs are calculated from the covariance matrix of the parameter estimates according to the method described by [4]. The code presented below is based on the `vif` function of the `rms` package [5] [6]. The function `vif.parf` (see below) has two arguments: `fit`, the fitted model (the `parfm` object) and `remove`, which contains the name of a variable equivalent to the intercept. In the case of a Weibull baseline `remove` is set to “`lambda`”.

```r
vif.parfm <- function (fit, remove = 'lambda') {

  if (class(fit)[1L] != "parfm")
    stop('The model must be fitted with parfm function')
  v <- attributes(fit)$varcov
  nam <- dimnames(fit)[[1L]]
  dimnames(v) <- list(nam, nam)

  # dropping equivalent of intercept given in the "remove" par
  pos <- sapply(remove, function(k) which(nam == k))
  if (length(pos)) {
    v <- v[-pos, -pos]
    nam <- nam[-pos]
  }

  d <- sqrt(diag(v))
  v <- diag(solve(v/(d %o% d)))
  names(v) <- nam
  v
}
```

References

1. Vaupel JW, Missov TI. Unobserved population heterogeneity: A review of formal relationships. Demographic Research. 2014;31: 659–686. doi:10.4054/DemRes.2014.31.22

2. R Core Team. R: A language and environment for statistical computing [Internet]. Vienna, Austria: R Foundation for Statistical Computing; 2019. Available: https://www.R-project.org/

3. Munda M, Rotolo F, Legrand C. parfm: Parametric frailty models in R. Journal of Statistical Software. 2012;51: 1–20. Available: http://www.jstatsoft.org/v51/i11/

4. Davis CE, Hyde JE, Bangdiwala SI, Nelson JJ. An example of dependencies among variables in a conditional logistic regression. Moolgavkar SH, Prentice RL, editors. New York: Wiley; 1986; 140–147.

5. Harrell FE. Rms: Regression modeling strategies [Internet]. 2019. Available: https://CRAN.R-project.org/package=rms

6. Harrell FE. Regression modeling strategies. Cham: Springer International Publishing; 2015. doi:10.1007/978-3-319-19425-7
